# Axial loading during MRI induces significant T2 value changes in vertebral endplates—a feasibility study on patients with low back pain

**DOI:** 10.1186/s13018-018-0727-z

**Published:** 2018-01-30

**Authors:** Hanna Hebelka, Andreia Miron, Izabela Kasperska, Helena Brisby, Kerstin Lagerstrand

**Affiliations:** 1 Department of Radiology, Sahlgrenska University Hospital, Gothenburg, Sweden; 2 Department of Orthopaedics, Sahlgrenska University Hospital, Gothenburg, Sweden; 3 Department of Medical Physics and Techniques, Sahlgrenska University Hospital, Gothenburg, Sweden; 4 Institute of Clinical Sciences Sahlgrenska Academy, University of Gothenburg, Gothenburg, Sweden

**Keywords:** Axial loading during MRI (alMRI), IVD, T2 mapping, Endplate

## Abstract

**Background:**

The function of the endplate (EP) is the most important factor influencing nutritional supply to the avascular intervertebral disc (IVD). It is desired to have a non-invasive method to assess functional EP characteristics in vivo. Assessment of functional EP characteristics is important in order to understand its relation to IVD degeneration, which in turn might deepen the understanding of the pathophysiology behind low back pain (LBP). It was hypothesized that, by comparing quantitative MRI of EPs performed with conventional supine MRI (unloaded MRI) with axial loading during MRI (alMRI), dynamical properties of the EP can be displayed. The aim was therefore to investigate the feasibility of axial loading during MRI (alMRI) to instantaneously induce quantitative EP changes.

**Methods:**

T2 mapping of 55 vertebral EPs (L1-S1) in five LBP patients was performed during conventional supine MRI (unloaded MRI) and subsequent alMRI. With T2 mapping, the cartilaginous EP and bony EP cannot be separated; hence, the visualized EP was termed EP zone (EPZ). Each EPZ was segmented at multiple midsagittal views, generating volumetric regions of interest. EPZs demonstrating signal inhomogeneity and/or adjacent Modic changes (MC) were termed abnormal EPZs. EPZ mean T2 values were compared between unloaded MRI and alMRI, and their relationship with abnormal EPZs was determined.

**Results:**

alMRI induced significantly higher (*p* = 0.01) EPZ mean T2 values compared with unloaded MRI. Significantly higher mean T2 values were seen in inferior EPZs compared with superior EPZs, both with unloaded MRI (35%, *p* < 0.001) and with alMRI (26%, *p* = 0.04). Significant difference between unloaded MRI and alMRI was seen in normal (*p* = 0.02), but not in abnormal EPZs (*p* = 0.5; *n* = 12).

**Conclusions:**

alMRI induces changes in human EPZ characteristics in vivo. The T2 value significantly increased in normal EPZs, with lack of such in abnormal EPZs. Combining T2 mapping with alMRI provides a clinical feasible, non-invasive method with potential to reveal biochemical behavioral patterns, thus adding another dimension of the EPZs characteristics compared with information obtained with solely unloaded MRI.

## Background

Low back pain (LBP) is an endemic condition with high impact for the individuals affected but also with tremendous socio-economic consequences [1]. LBP is closely associated with both degeneration of the intervertebral disc (IVD) and with vertebral endplate (EP) changes [2–5]. The exact etiological factors for LBP are however still not elucidated and neither are the exact mechanisms initiating the degenerative cascade of the IVD. Further, the lack of a true “gold standard” diagnostic tool that unequivocally points out which spinal structures cause LBP prevents optimal success for used and suggested therapeutic strategies [6, 7].

The avascular IVD relies on passage through the EP for nutrient supply and metabolite removal, primarily regulated by diffusion and convection via the vertebral EP [3, 8]. Dynamic contrast-enhanced MRI (DCE-MRI) has revealed that EPs with signs of degeneration have altered contrast enhancement pattern compared to non-degenerated EPs [9–11]. Hence, structural EP changes, like fissures or sclerosis, may alter its permeability and play a crucial role in the progression of IVD degeneration [2, 3, 8, 12]. The exact relationship between compromised EP function and IVD degeneration is not fully understood. To deepen the understanding of the pathophysiology behind IVD degeneration and, in extension, also the understanding of LBP, non-invasive diagnostic tools with ability to in detail characterize early biochemical EP changes are desirable.

Quantitative MRI methods, sensitive for alterations in composition between macromolecules, collagen, and water, reliably reflect changes in biochemical composition and structural integrity in degenerated IVDs [13–16]. In spite of the ability of these methods to reveal early signs of IVD degeneration and thereby potentially find the window when biological targeted therapy might be favorable, quantitative MRI methods are still not used routinely in a clinical setting. Quantitative MRI studies of the EPs are, in contrast to the above discussed IVD studies, limited but have been reported to also reveal subtle deterioration of biochemical EP composition [17–19].

Mechanical loading of the spine has been shown to affect quantitative MRI parameters in studies investigating the IVDs [15, 16, 20–22]; however, similar studies of the EPs are lacking. A recent review regarding the immediate lumbar response to various loading conditions concluded that there is a gap in knowledge on how loading affects spinal imaging [23]. Imaging of the EP in a loaded state versus in an unloaded state might reveal differences in EP characteristics that may reflect altered EP functionality. It was hypothesized that by comparing quantitative MRI of EPs performed with conventional supine MRI (unloaded MRI) with axial loading during MRI (alMRI), dynamical properties of the EP can be displayed. The aim of the present study was to investigate if alMRI instantaneously can induce EP changes, measured with quantitative MRI.

## Methods

### Study participants

Fifty-five vertebral EPs were investigated in five LBP patients (three female/two male) with mean age 37 years (range 28–49). The patients were recruited, consecutively, among patients included in an ongoing, large-scale LBP study. All patients, referred from the spine surgery unit at Sahlgrenska University Hospital, Gothenburg, Sweden, declined any radicular symptoms, and none had undergone previous disc surgery or had any contraindications for performing MRI.

### Magnetic resonance imaging

Each participant was examined with a spinal magnetic resonance imaging (MRI) Siemens Magnetom Aera 1.5T scanner (Erlangen, Germany), consisting of an initial conventional, supine protocol (unloaded MRI) (20 min) followed by an identical repeat supine protocol with the addition of axial loading (alMRI) (20 min). The T2 mapping sequence was included last in the protocols; hence, the T2 mapping with and without axial load was performed for 20 min apart. The alMRI was performed in supine position with a Dynawell compression device (Dynawell diagnostics AB, Las Vegas, NV, USA) with 50% of the patient’s body weight applied to simulate the load in upright position [20]. The protocol constituted of TSE T1- and T2-weighted (W) sequences in the sagittal plane, an axial TSE T2W sequence and a SE T2 mapping sequence in the sagittal plane (Table 1). Calculation of T2 value voxel by voxel was based on multi-parametric model.

**Table 1:** MRI and alMRI protocol details

	NSA (*n*)	Flip angle	TR (ms)	TE (ms)	Matrix	FOV (cm)
TSE T1 sag	2	150	480	9	320 × 320	30 × 30
TSE T2 sag	1	150	3500	95	384 × 384	30 × 30
TSE T2 ax	2	150	5330	97	320 × 320	20 × 20
SE T2-map	1	150	1400	11–89 (*n* = 8)	256 × 256	22 × 22

### Measurements

**Fig. 1:**
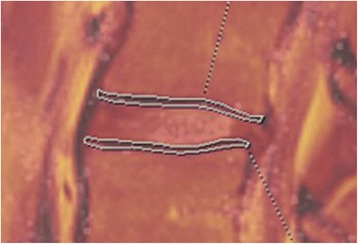
Illustration of endplate zone (EPZ) segmentation. Illustration of endplate zone (EPZ) segmentation of two EPZs in midsagittal view, on fused T1-weighted and T2 map images respectively

Since the cartilaginous EP and the bony EP cannot be separated with T2 mapping, the outlined vertebral EP was termed the EP zone (EPZ) (Fig. 1). Since T1 signal overlay on the T2 mapping images improved visibility of the EPZ, segmentation of the EPZ was made on fused sagittal T1 and T2 mapping sequences on the Syngovia (Siemens) image processing software (Fig. 1). To achieve volumetric delineation, the images were reformatted into non-overlapping 10-mm slices. Segmentation was performed on three consecutive 10-mm slices, one midsagittal and two parasagittal. In this fashion, one volumetric segmentation of each EPZ was finally obtained, covering 30 mm of the median EPZ width in total. Mean T2 values (ms) and standard deviations (SD) of the means (ms) and areas (mm^2^) were recorded for the whole volume. The measurements were repeated in identical fashion on alMRI images.

Twenty-five IVDs were graded, on T2W sequences, according to Pfirrmann classification [24], with grades 1–2 considered as non-degenerated IVDs while grades 3–5 were considered degenerated.

On the unloaded MR images, each vertebra (L1-S1) was divided in 50% in cranio-caudal direction to obtain vertebral units directly adjacent to each superior and inferior EP respectively. In each vertebral unit, any existence of Modic changes (MC) were registered and graded as 0 (no changes) and 1 (any MC type) respectively [5]. In addition, visually observable EPZ changes, like Schmorl’s nodules or signal inhomogeneity (i.e., discontinuity of the EPZ, fissures, inhomogeneous signal), in each EPZ were evaluated and graded as 0 (no changes) or 1 (signal inhomogeneity). If signal inhomogeneity or MC were detected anywhere in the EPZ (30 mm width), it was considered as a positive finding. To term EPZs with either signal inhomogeneity or adjacent to MC, the term abnormal EPZs will be used while EPZs without any of these will be termed normal EPZs. It was also registered whether the EPZ was a superior or inferior vertebral EPZ.

All measurements were made by a radiology resident (AM) who also performed intra-observer analysis after 3 months, blinded to the original results. Blinded inter-observer analysis was performed by a senior radiologist with over 13 years’ experience in the spinal field (HH).

### Statistical analysis

Descriptive data are expressed in terms of mean and standard deviation (SD). A parametric paired *t* test was used for comparison between differences in dependent T2 values between unloaded MRI and alMRI for the various parameters investigated. Fisher’s exact test was used for calculation of the relation between Pfirrmann grade and EP changes. Reliability of quantitative T2 value measurements for both intra- and inter-rater agreement was performed using intraclass correlation coefficient (ICC), model 2, with 95% confidence intervals. The coefficients were interpreted using Cronbach’s alpha [25]. All tests were two-sided, and statistical significance was set as *p* < 0.05. Data was analyzed using IBM SPSS Statistics for Windows, Version 22.0. (IBM Corp, Armonk, NY, USA).

## Results

### Comparison of all EPZs between unloaded MRI and alMRI

**Fig. 2:**
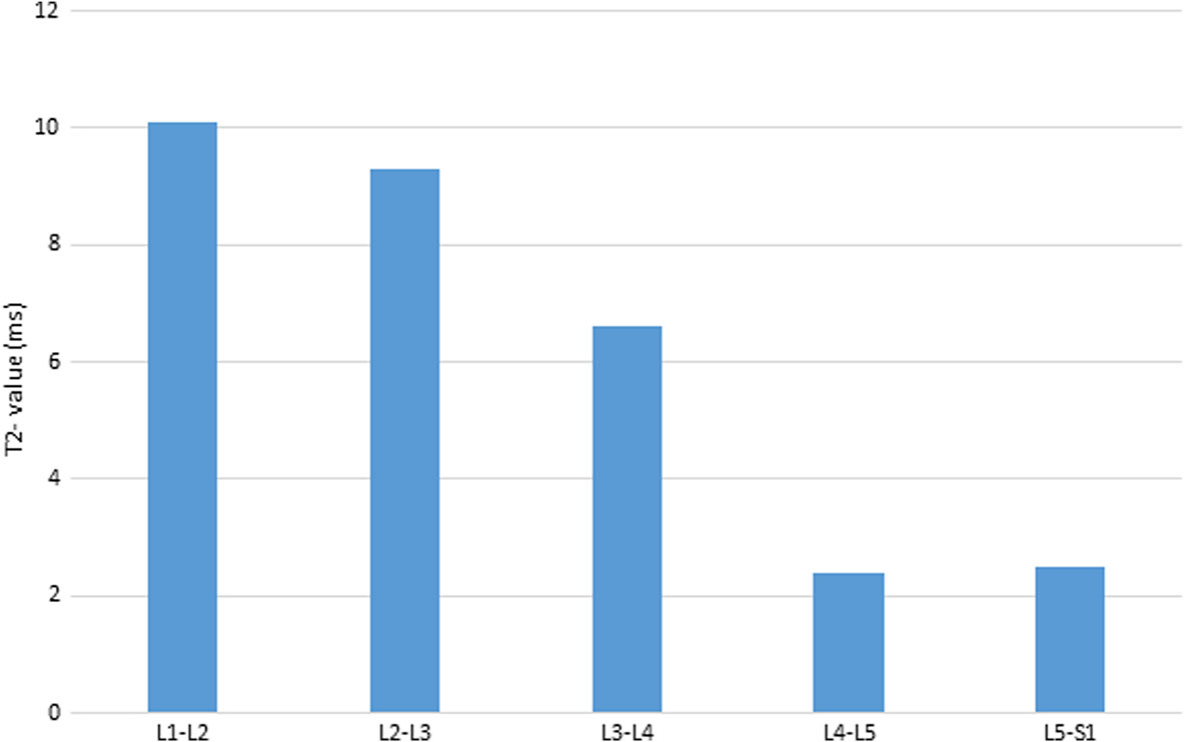
Mean endplate zone (EPZ) T2 value change for each lumbar level. Difference in endplate zone (EPZ) mean T2 values (ms) between unloaded MRI and alMRI for each lumbar level

The alMRI induced significantly higher mean T2 values in the EPZs compared with unloaded MRI, with a mean increase of 19% (*p* = 0.01) (Table 2). When analyzed on disc level, a trend towards reduced differences induced was seen in caudal direction of the lumbar spine (Fig. 2).

**Table 2:** T2 values (ms) of endplates zones (EPZ) with unloaded MRI (uMRI) and alMRI

	*N*	Minimum	Maximum	Mean	SD	*p* value
EPZ uMRI	55	22	75	36	12	0.01
EPZ alMRI	55	21	80	43	17	
Inferior EPZ uMRI	25	23	75	42	15	< 0.001
Superior EPZ uMRI	30	22	49	31	7	
Inferior EPZ alMRI	25	25	80	48	17	0.04
Superior EPZ alMRI	30	21	79	38	16

### Comparison between superior and inferior EPZs

Significantly higher mean T2 values were seen in inferior EPZs compared with superior EPZs, both with unloaded MRI (35%, *p* < 0.001) and with alMRI (26%, *p* = 0.04) (Table 2).

### EPZ abnormalities and IVD degeneration

One L5-S1 IVD was sacralized with only a rudimentary IVD. Of the remaining 24 IVDs, none had Pfirrmann grade 5 and only one had grade 1. In total, eight IVDs were classified as degenerated and 16 non-degenerated. Signal inhomogeneity was detected in 11 EPZs, of which six were adjacent to MCs and nine (82%) bordered to degenerated IVDs; MCs (all type 1) were found adjacent to seven EPZs. In total, 12 abnormal EPZs were registered.

### Comparison between induced EPZ changes in normal EPZs versus abnormal EPZs

A significant difference between unloaded MRI and alMRI (from 36 to 43 ms; *p* = 0.03) was found in EPZs without signal inhomogeneity. In EPZs with signal inhomogeneity, no such difference was seen (from 38 to 41 ms; *p* = 0.3). In EPZs adjacent to vertebras without MC (*n* = 48), alMRI induced an increased mean T2 value (from 36 to 43 ms; *p* < 0.02). In EPZs adjacent to MCs, no increment in the T2 value was registered (from 42 to 39 ms; *p* = 0.3). Significant difference between unloaded MRI and alMRI was seen in normal (*p* = 0.02), but not in abnormal EPZs (*p* = 0.5; *n* = 12).

**Fig. 3:**
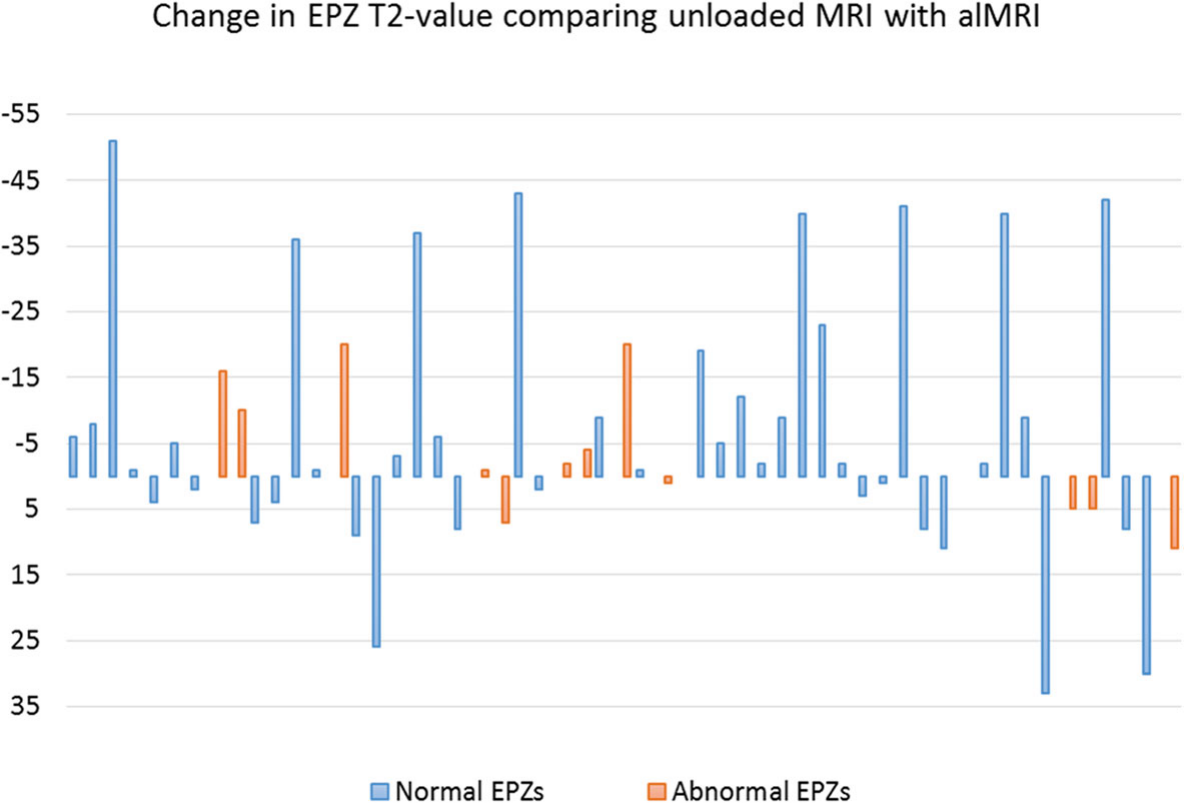
T2 value changes for each of the 55 endplate zones (EPZs). T2 value changes for each of the 55 EPZs between unloaded MRI and alMRI. An increase in T2 values is thus displayed as negative values and a decrease displayed as positive values

The variation in the T2 value was however large for both normal and abnormal EPZs (Fig. 3). With unloaded MRI, normal EPZs displayed a higher variation (CV = 36%) compared with abnormal EPZs (CV = 30%). With alMRI, the variation in the T2 value increased for normal EPs (CV = 42%), however, not for abnormal EPZs (CV = 29%).

### Reliability measurements

Reliability measurements of EPZs T2 value showed very good agreement with Crohnbach’s alpha 0.86 (95% CI 0.79–0.91) for intra-rater measurements and 0.85 (95% CI 0.78–0.91) for inter-rater agreement respectively.

## Discussion

This feasibility study, comparing T2 values of EPZs obtained by unloaded MRI and alMRI, showed that significantly higher T2 values were induced in the loading state. In abnormal EPZs, no significant induced difference was shown at a group level as opposed to in normal EPZs. Further, the known morphological and functional differences, like for example different stiffness and ability to resist load [26], between superior and inferior EPs were clearly displayed quantitatively.

Spinal load has been shown to affect quantitative MRI parameters of the IVD, which may provide detailed information of the IVD characteristics and its behavioral patterns [15, 16, 20–22]. In this study, it was hypothesized that alMRI would also affect quantitative EP values. The present study seems to confirm this hypothesis by displaying significant induced changes in normal EPZs, however, not in abnormal EPZs. Hence, combining T2 mapping with alMRI adds another dimension of the EPZ characteristics compared with information obtained with solely unloaded MRI and may, thus, provide a clinical feasible, non-invasive method with potential to reveal biochemical behavioral patterns of the EPZs.

The cartilaginous EP consists of approximately 50–60% water [18], with a high degree of transport in the well-hydrated matrix. With degeneration, the permeability of the EP decreases due to occlusion of the vascular buds and increased ratio of calcification and collagen content [18, 27, 28] which restrict the nutritional transport over the EP [8, 9, 27]. EP damage can however paradoxically also result in increased permeability with loss of essential nutrients and enzymes [9]. For example, Rajesekaran et al. performed serial post contrast-enhanced MRI sequences in 54 individuals and reported either enhanced diffusion or delayed such as in EPs with degenerative signs (fissures, Schmorls, etc.), showing that type of EP injury results in different functional behavior [8, 9, 27]. In general, higher T2 values are interpreted as high hydration. However, this is a simplified model since T2 values reflect not only the composition between proteoglycans, collagen, and water but also depend on tissue anisotropy, i.e., matrix organization [14, 15, 21, 22, 29, 30]. Why certain EPZs respond to load in different ways are obviously complex and probably multifactorial. The differences between abnormal and normal EPZs seen in the current study show, however, that the EPZs display different characteristics, and the lack of response from alMRI in abnormal EPZs may be a reflection of impaired integrity.

Despite the fact that alMRI induced an increase in the EPZ T2 value on a group level, on an individual EPZ-level, a large variation in the induced changes were seen, from 187% increase to 55% decrease (Fig. 3). In abnormal EPZs, the range of induced changes was not as pronounced (89 and 26% respectively). This wide range of T2 values may in fact reflect large differences in the characteristics between individual EPZs, and the smaller range of values in abnormal EPZs may reflect a lower dynamical variation due to impaired EP function. Future studies are warranted to strengthen these results and to investigate the pathophysiological mechanisms behind this divergent behavior between normal and abnormal EPZs.

Mechanical stimuli can exert both anabolic and catabolic effects on the spine [17, 29–31]. The effect that alMRI exert on each EP is not known, and it cannot be excluded that this instantaneous load do not impair EP transport at some levels. However, since alMRI aims to simulate the load under upright position, alMRI have been assumed to exert load within a physiological beneficial range [32]. Other potential factors that might influence the various alMRI-induced EPZ responses are the level of the EPZ in the lumbar spine, the lordosis and IVD angle induced, and the status of the EPZ and IVD. Recently, alMRI was shown to induce regional alterations in IVD T2 values depending on IVD level [20]. The present feasibility study was not powered to investigate such relations. Induced differences however seemed to decline in caudal direction of the lumbar spine, which speculatively might be explained by degenerative changes being more common caudally. Out of 16 degenerated IVDs, 12 were localized at L4-S1 level as were nine of the 12 abnormal EPZs.

The absolute T2 values obtained in the present study were slightly lower (22–74 ms unloaded MRI) than previously reported by Delucca et al. (60–100 ms) [28], a discrepancy likely due to either the previous cadaver study design or that outlining of only the cartilaginous EP was not possible in the current in vivo study. The significant difference in absolute T2 values between superior and inferior EPZs likely reflects what has been shown in biomechanical in vitro studies and animal in vivo with the inferior EP being stiffer, resist load better and with different transport kinetics compared with the superior EP [26, 30]. This phenomenon is reflected in clinical situations where for example superior EPs being more prone to burst fractures [33]. Also human in vivo studies show that contrast enhancement differ between superior and inferior vertebral EPs [10, 11]. To our knowledge, this is the first human in vivo study displaying quantitative differences, which may reflect morphological differences, between the superior and inferior EPZs. Thus, these results constitute ground data for future studies.

Data from a single time point cannot fully characterize the complex kinetics occurring in the EP and why comparing induced changes between unloaded MRI and alMRI provides another dimension, indicating dynamic EP characteristics. Such has been reported promising in the IVD [20], and this study shows that alMRI is feasible to induce changes also in the less hydrated EP. Despite large differences at individual levels, significant differences were found at group level and in addition with a strong test-retest repeatability, making the results of this feasibility study worth studying in large-scale studies.

The distinct responses induced with alMRI makes the combination of quantitative MRI and alMRI a promising method to assess behavioral EP characteristics clinically. In addition, alMRI offers a method to image the spine in a position inducing concordant pain in LBP patients [34]. To elucidate if the differences in EPZ characteristics between unloaded MRI and alMRI represent altered diffusion/perfusion, future studies are encouraged to include diffusion-weighted sequences. If this method can reveal also early signs of impaired EP integrity, before manifest IVD degeneration appears, it would provide important information that ultimately may be used for targeted therapy. To evaluate clinical application of the method, work has been initiated to elucidate if this method can depict pain predictors by comparing if the induced EP T2 value changes vary between a large-scale LBP cohort and healthy controls.

The small sample size is a limitation. In a previous alMRI study that investigated IVD changes [20] (50 IVDs), significant changes were reported, why the current sample size was considered appropriate to evaluate if it is feasible to induce EPZ T2 value changes with alMRI. However, considering the limited number (*n* = 12) of abnormal EPZs, it cannot be excluded that this study is underpowered for evaluation of behavioral pattern regarding abnormal EPZs. The so-called “magic angle effect,” appearing at approximately 55° from the main magnetic field [18], must be considered. However, the effect in the current study is regarded as non-existing at L1-L4 and only minor at L5-S1 since none of the EPZs at level L1-L4 were imaged between 50° and 60° with unloaded MRI and only one L5 EPZ was imaged at 58° and four S1 EPZs imaged at 53°–54°. With alMRI only, three EPZs were imaged between 50° and 60°.

Contribution of minor signal from adjacent tissue, for example the IVD, cannot be completely excluded due to the image resolution obtained with T2 mapping. However, this potential partial volume effect ought to be minor on the whole segmented EPZ volume and in addition negligible regarding induced EPZ changes since such effect ought to be similar on unloaded MRI and alMRI.

## Conclusion

This study shows, for the first time, that alMRI induce changes in human EPZs characteristics in vivo. However, when analyzed on group level, the T2 value significantly increased in normal EPZs, with lack of such in abnormal EPZs. Combining T2 mapping with alMRI provides a clinical feasible, non-invasive method with potential to reveal biochemical behavioral EP patterns, thus adding another dimension of the EPs characteristics compared with information obtained with solely unloaded MRI. Thus, this study opens up an area for future research since imaging during loading conditions might increase the diagnostic specificity, providing new biomarkers, within spine imaging.

## Abbreviations

alMRI: Axial loading during MRI
EP: Endplate
EPZ: Endplate zone
ICC: Intraclass correlation coefficient
IVD: Intervertebral disc
LBP: Low back pain
MC: Modic changes
MRI: Magnetic resonance imaging
